# Combination of treosulfan, fludarabine and cytarabine as conditioning in patients with acute myeloid leukemia, myelodysplastic syndrome and myeloproliferative neoplasms

**DOI:** 10.1007/s00432-021-03836-8

**Published:** 2021-10-21

**Authors:** Samantha O‘Hagan Henderson, Jochen J. Frietsch, Inken Hilgendorf, Andreas Hochhaus, Claus-Henning Köhne, Jochen Casper

**Affiliations:** 1 Onkologie und Hämatologie, Universitätsklinikum Oldenburg, Klinik Für Innere Medizin II, Oldenburg, Germany; 2 Abteilung Hämatologie und Internistische Onkologie, Universitätsklinikum Jena, Klinik Für Innere Medizin II, Am Klinikum 1, 07747 Jena, Germany

**Keywords:** AML, MDS, MPN, Allogeneic stem cell transplantation, Toxicity-reduced conditioning

## Abstract

**Purpose:**

Treosulfan and fludarabine (Treo/Flu) were successfully introduced into toxicity-reduced conditioning for SCT. However, the risk of post-SCT relapse remains a matter of concern. We report the results of a novel individual treatment approach with Treo/Flu and cytarabine (Treo/Flu/AraC) conditioning prior to allogeneic SCT in patients with acute myeloid leukemia (AML), myelodysplastic syndrome (MDS), or myeloproliferative neoplasms (MPN).

**Methods:**

Seventy-seven patients (median age 54 years) at high risk of disease relapse due to unfavorable cytogenetics or failure to achieve complete remission prior to SCT were included. Median follow-up was 3.2 years.

**Results:**

The 1-, 2- and 3-year RFS rates were 49.4%, 41.7%, and 37.6% and OS rates were 59.3%, 49.3%, and 45.4%, respectively. Cumulative incidence of NRM was 10% at 100 days, 18.8% at 1 year and 20.1% at 2 years. The cumulative incidence of relapse increased from 31% at 1 year to 38.5% after 3 years. The cumulative incidences of engraftment, chimerism, graft-versus-host disease (GvHD) and toxicities were acceptable and comparable with similar patients conditioned with Treo/Flu or FLAMSA-RIC.

**Conclusion:**

In conclusion, Treo/Flu/AraC provides tolerable, feasible, and effective conditioning for patients with AML, MDS or MPN, even in advanced disease states. The incidence of NRM and relapse is acceptable in this heavily pre-treated population with high-risk disease. Future research will aim to confirm these initial findings and include a larger number of participants in a prospective trial.

**Supplementary Information:**

The online version contains supplementary material available at 10.1007/s00432-021-03836-8.

## Purpose

Patients with active disease before allogeneic stem cell transplantation (SCT) or high risk of relapse are faced with a poor prognosis. The intensity of conditioning can heavily influence patient outcome. Myeloablative protocols reduce the risk of disease relapse compared to reduced intensity conditioning (RIC), but high non-relapse mortality (NRM) rates are a concern (Scott et al. 2017). RIC has made allogeneic SCT accessible to those previously deemed unfit.

The first study to demonstrate the feasibility of conditioning with treosulfan and fludarabine (Treo/Flu) was published in 2004 (Casper et al. 2004a). Treosulfan is an alkylating agent with acceptable extra-medullary toxicity at doses up to 46 g/m^2^ (Scheulen et al. 2000). It has pronounced in vitro committed and non-committed hematopoietic stem cell toxicity (Beelen et al. 2005) and in vitro anti-leukemic cell activity (Munkelt et al. 2008). Fludarabine is a purine analog that inhibits DNA and RNA synthesis (Gandhi and Plunkett 2002). Fludarabine has replaced cyclophosphamide in many conditioning regimens due to its improved toxicity profile (Ben-Barouch et al. 2016).

Treo/Flu conditioning has been introduced in the management of patients with AML, MDS, and MPN (Hilgendorf et al. 2011; Casper et al. 2010; Schmidt-Hieber et al. 2007; Kroger et al. 2006; Shimoni et al. 2010). The European Society for Blood and Marrow Transplantation (EBMT) registry data were used to compare Treo/Flu with other conditioning regimens such as FLAMSA-RIC (fludarabine, amsacrine and cytarabine) and busulfan-based regimens in patients with AML or MDS. Lower rates of acute graft-versus-host disease (aGvHD) were seen in those treated with Treo/Flu (Shimoni et al. 2018; Sheth et al. 2019). However, when Treo/Flu was compared to FLAMSA-TBI, the latter had a decreased risk of relapse and better leukemia-free survival (Sheth et al. 2019). A recent randomized phase III clinical trial compared the outcomes of patients with AML or MDS undergoing allogeneic SCT with either Treo/Flu or Bu/Flu conditioning. This study demonstrated the non-inferiority of Treo/Flu compared to Bu/Flu (Beelen et al. 2020). Two-year overall survival (OS), transplant-related mortality, and NRM were all significantly better after Treo/Flu conditioning. Event-free survival was better in the Treo/Flu group, but did not reach significance.

Cytarabine, a pyrimidine analog, is a chemotherapeutic backbone in the treatment of hematologic malignancies. It is one of the most effective drugs for the treatment of AML, even after disease relapse (Capizzi 1996; McLaughlin et al. 2012). When fludarabine is infused prior to the administration of cytarabine, the intracellular accumulation of the biologically active form of cytarabine is potentiated (Gandhi et al. 1993).

By combining cytarabine, an effective drug in the treatment of myeloid malignancies, with a well-tolerated and effective reduced toxicity conditioning regimen (Treo/Flu), the aim was to achieve better outcomes for patients transplanted with active disease and those with a high risk of relapse. The Treo/Flu/AraC regimen has potentially very strong anti-leukemic, immunosuppressive and cytotoxic effects. This retrospective study evaluates the outcomes of patients with AML, MDS, or MPN following conditioning with Treo/Flu/AraC in comparison to Treo/Flu prior to allogeneic SCT to evaluate the additive effect of AraC.

## Patients and methods

Seventy-seven patients (32% female) with AML, MDS, and MPN between 18 and 69 years of age (median 54 years) were conditioned with Treo/Flu/AraC (see Table 1 for patient and donor characteristics) between July 2009 and August 2018 at the University Hospitals of Jena and Oldenburg in Germany. Patients were followed up until June 2019. The decision to give reduced-intensity conditioning with Treo/Flu/AraC was based on patient-specific factors including comorbidities, previous treatments received, previous response to therapy, and disease remission status. Of note, ten patients had received previous allogeneic SCT and three of those Treo/Flu/AraC conditioning at the time of the first and second transplant. All patients gave written informed consent.

**Table 1:** Characteristics of all patients and donors

Patient or graft (donor) characteristic	Number (percentage)
Patient age at transplant median (range), years	54 years (18–69 years)
Patient’s sex	
Male	52 (68%)
Female	25 (32%)
Female donor for male recipient	17 (21%)
Diagnosis at time of transplantation	
Primary acute myeloid leukemia	33 (43%)
Secondary acute myeloid leukemia	25 (32%)
Myelodysplastic syndrome	6 (8%)
Myeloproliferative neoplasm	13 (17%)
Primary AML ELN risk stratification by genetics*	
Favorable	5 (15%)
Intermediate	20 (61%)
Adverse	8 (24%)
Status at transplantation	
CR1	19 (25%)
CR2	13 (17%)
≥ CR3	3 (4%)
First or second partial response	11 (14%)
Relapse	6 (8%)
Progressive disease	13 (17%)
First or second chronic phase	5 (6%)
First blast crisis	1 (1%)
Stable disease	6 (8%)
CR1 (breakdown of diagnoses within this group)	
Secondary AML	9
Primary AML	8
Adverse genetic risk (ELN)*	3
Intermediate genetic risk (ELN)*	4
High-risk CML (ELTS score)	3
Treatment before transplantation (no. of courses)	
None	2 (3%)
One	24 (31%)
Two	30 (39%)
> Two	21 (27%)
Previous allogeneic SCT	10 (13%)
CMV status (patient/donor)	
Negative/negative	24 (31%)
Negative/positive	9 (12%)
Positive/negative	17 (22%)
Positive/positive	27 (35%)
Donor	
Matched-related donor (MRD)	20 (26%)
Matched-unrelated donor (MUD)	47 (61%)
Mismatched-unrelated donor (MMUD)	10 (13%)
Stem cell source	
Bone marrow	2 (3%)
Mobilised peripheral blood stem cells	75 (97%)
Hematopoietic cell transplantation-specific comorbidity index (HCT-CI)	
0	46 (60%)
1–2	20 (26%)
> 2	11 (14%)

*AML* acute myeloid leukemia, *CMV* cytomegalovirus, *CR* complete remission, *CR1/2/3* first/second/third complete remission, *ELN* European LeukemiaNet, *ELTS* EUTOS long-term survival, *HCT-CI* Hematopoietic Cell Transplantation-specific Comorbidity Index, *MMUD* mismatched-unrelated donor, *MRD* matched-related donor, *MUD* matched-unrelated donor, *SCT* stem cell transplantation

*Döhner et al. 2017

Fifty-two patients received treosulfan (Medac, Wedel, Germany) 14 g/m^2^ intravenously over 2 h from day −6 to −4. Twenty-eight patients received treosulfan on day −4 to −2. Fludarabine (Schering, Berlin, Germany) 30 mg/m^2^ was given intravenously over 30 min from day −6 to −2. Cytarabine (various manufacturers) was administered at a dose of 2000 mg/m^2^ once daily over 3 h on day −6 to −5. Rabbit antithymocyte globulin (Neovii, Graefelfing, Germany) was given only for patients receiving grafts from unrelated donors as previously described (Casper et al. 2004a).

### Supportive care

Supportive care was given based on local guidelines. Prophylaxis against GvHD, consisting of cyclosporin A (CsA) and methotrexate, has been described elsewhere (Casper et al. 2012). Whole-blood steady-state trough concentrations of CsA were maintained between 180 ng/ml and 230 ng/ml. Based on its positive effect on patients’ survival, ursodeoxycholic acid was administered daily to patients with rising serum bilirubin levels and in patients suspected of developing GvHD (Ruutu et al. 2014).

### Chimerism

Chimerism was evaluated using PCR to amplify previously identified microsatellites in DNA extracts of peripheral blood mononuclear cells. Patients were classified as a complete chimera if the proportion of donor cells exceeded 98%. Chimerism analysis was performed on day + 28, + 100 and + 180.

### Definition of primary and secondary outcomes and statistical analysis

The primary end point was relapse-free survival (RFS) and this was calculated for each transplantation. The definition of RFS and analysis has been described previously (Hilgendorf et al. 2011).

Secondary end points were OS, NRM, cumulative incidence of relapse, engraftment and graft failure, chimerism, acute or chronic GvHD (aGvHD, cGvHD), and toxicities/adverse events as defined by the Common Terminology Criteria for Adverse Events (CTCAE) version 5.0. Toxicities and adverse events were recorded during conditioning until day + 28.

OS was calculated from day 0 to death due to any cause and was analyzed using the Kaplan–Meier method. Patients alive at their last follow-up were censored. NRM, relapse, engraftment, chimerism, and aGvHD or cGvHD were estimated using cumulative incidence analysis considering competing risks. NRM was defined as death without previous relapse of disease. Relapse was a competing event. NRM was a competing event with regard to relapse. For the three patients who received two transplantations with the Treo/Flu/AraC regimen, RFS and relapse were calculated from the time of the second transplantation. Engraftment of neutrophils and platelets was defined as described previously (Casper et al. 2010). Primary graft failure (Olsson et al. 2013), poor graft function (Larocca et al. 2006), and secondary graft failure (Olsson et al. 2015) have also been described elsewhere. Acute and chronic GvHD was classified according to the criteria of Harris et al. (2016) and Filipovich et al. (2005), respectively.

For exploratory purposes, outcome data (RFS, OS and NRM) were stratified by type of donor, remission status at transplantation (CR vs. all other statuses) and age (< 50 years vs. ≥ 50 years). In the statistical analysis comparing groups, log-rank tests were used in Kaplan–Meier analyses and Gray’s test was applied to cumulative incidence curves. A subgroup analysis of AML patients was also performed.

Median follow-up time was calculated using the method described by Schemper and Smith (1996).

Statistical analysis using the Kaplan–Meier method was performed using SPSS (IBM Corp. 2017. Version 25.0. Armonk, NY, USA). Cumulative incidence curves with competing risk analysis and 95% confidence intervals were performed using R version 3.5.3 provided by the R Foundation. The method has been described elsewhere (Scrucca et al. 2007). Statistical significance was defined at 0.05.

### Ethics and data protection

The ethics committees of the Universities of Oldenburg and Jena approved the study in its current form (reference number Oldenburg: 2018–106, Jena: 2019–1316-BO). Analysis was performed on anonymized data and in accordance with the Declaration of Helsinki.

## Results

At the time of allogeneic SCT, the majority of patients (55%, 42/77) were not in remission. The median percentage of bone marrow blasts at the start of conditioning was 22.6% [CI (15–30%)] in these patients and 12 of those patients harbored the following mutations: FLT3-ITD (3), FLT3-ITD and NPM1 (2), FLT3-ITD, FLT3-TKD and MLL (1), FLT3-TKD and MLL (1), IDH1, U2AF1 and MPL (1), MLL (1) and NPM1 (1). In contrast, 16 of the patients in CR harbored the following aberrations: FLT3-ITD (5), FLT3-ITD and NPM1 (6), FLT-TKD (1), NRAS and U2AF1 (1), RUNX1 (1), RUNX-RUNX1T1 and FIP1L1-PDGFRA (1), RUNX-RUNX1T1 and FLT3-ITD (1). Median follow-up time was 3.2 years (range 13 days – 9.8 years) after SCT.

### Relapse-free survival (RFS)

**Fig. 1:**
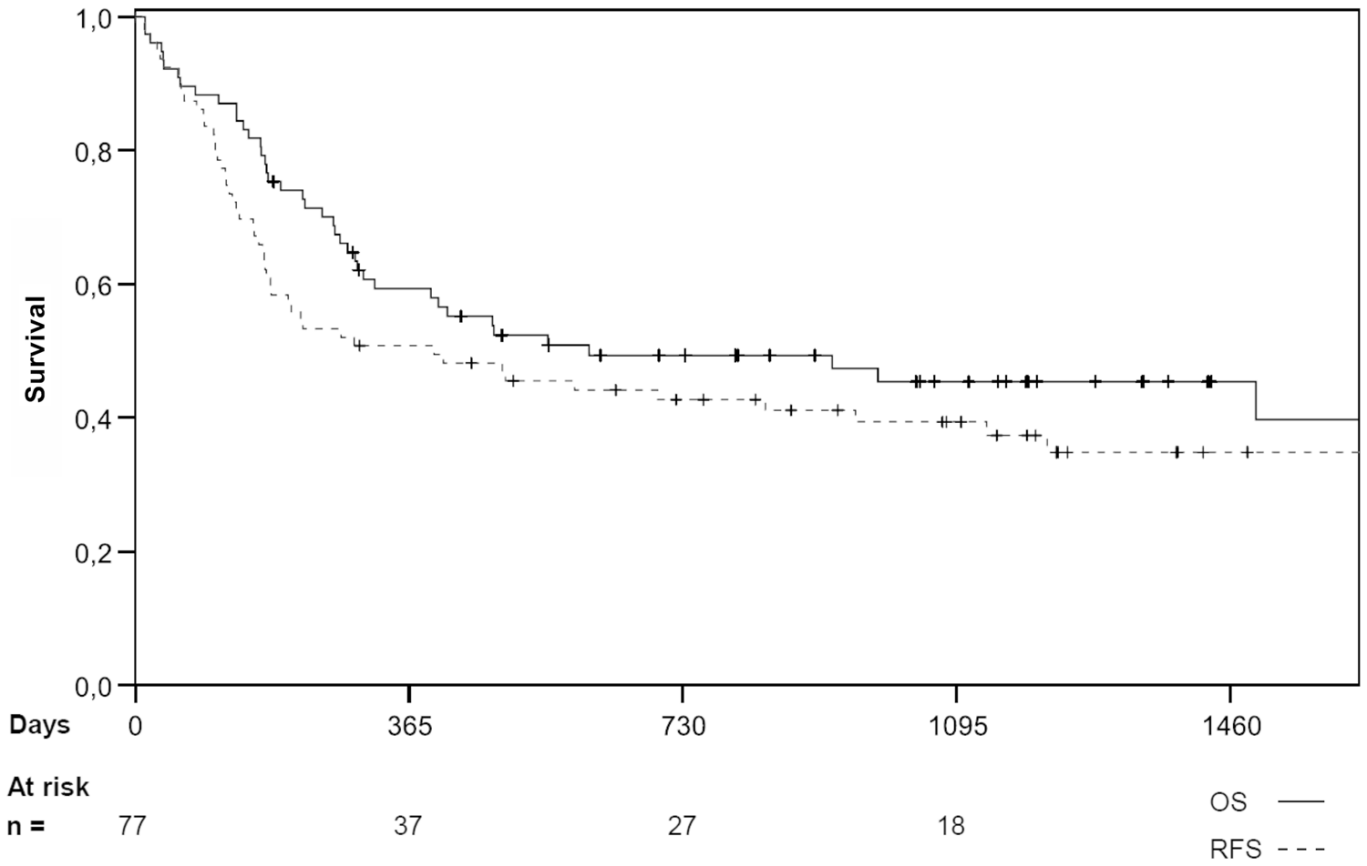
Overall survival estimates (full line) and relapse-free survival estimates (broken line), calculated using the Kaplan–Meier method, for all 77 patients conditioned with Treo/Flu/AraC. Small vertical lines denote a censored event

The 1-, 2- and 3-year RFS was 49.4%, 41.7%, and 37.6% (see Fig. 1). For matched unrelated donor (MUD) transplantations, these figures were 48.9%, 39.7%, and 37.0%, and for matched-related donor (MRD) transplantations, 55.0%, 49.5% and 41.3%, respectively. For mismatched-unrelated donor (MMUD) transplantations, RFS was 40.0% at 1 year and remained at this level throughout follow-up. There was no significant difference between the groups (*p* = 0.74).

Patients transplanted in CR had a 1-, 2- and 3-year RFS of 55.1%, 48.2%, and 43.8%. The figures for those transplanted in non-CR were 50.2%, 45.5%, and 43.6% (*p* = 0.992).

There was no significant difference in the RFS between patients receiving a transplant at or over the age of 50 (1-, 2-, and 3-year RFS was 50.0%, 43.9%, and 41.0%, respectively) when compared with patients younger than 50 years old (48.0%, 40.0%, and 36.0%; *p* = 0.966) (see supplemental table S1).

The 1-, 2- and 3-year RFS rates for the AML subgroup analysis were 43.3%, 38.1%, and 33.5%.

### Overall survival (OS)

The 1-, 2- and 3-year OS was 59.3%, 49.3%, and 45.4% (see Fig. 1). For MRD transplantations, these figures were 70.0%, 48.1% and 40.1%, and for MUD transplantations, these figures were 56.8%, 49.9%, and 46.7% (*p* = 0.95). The 1-year OS for those receiving a MMUD was 48.0%.

For patients transplanted in CR, the 1-, 2- and 3-year OS was 65.6%, 53.3%, and 45.1%, and for those transplanted not in CR 54.3%, 46.2%, and 46.21% (*p* = 0.996), respectively (see supplemental table S2). The 1-, 2- and 3-year OS for patients aged under 50 was 58.1%, 54.2%, and 44.4% and for patients aged 50 and over 60.3%, 47.4%, and 44.5% (*p* = 0.785), respectively.

The 1-, 2- and 3-year OS of the patients with AML was 56.5%, 48.9%, and 43.5%.

### Non-relapse mortality and cumulative incidence of relapse

Eighteen patients were neutropenic before the start of conditioning and eight received conditioning despite radiological evidence of fungal pneumonia. Eighteen patients died without disease relapse. The causes of death of those patients who died before day + 28 were: sinusoidal obstruction syndrome (1), sepsis (2) or pneumonia (1). The causes of death for the remaining 14 patients were: sepsis/infection (7), acute respiratory distress syndrome (2), intracerebral bleed (1), post-transplant lymphoproliferative disorder (1), grade IV liver GvHD (1), or unknown (2).

**Fig. 2:**
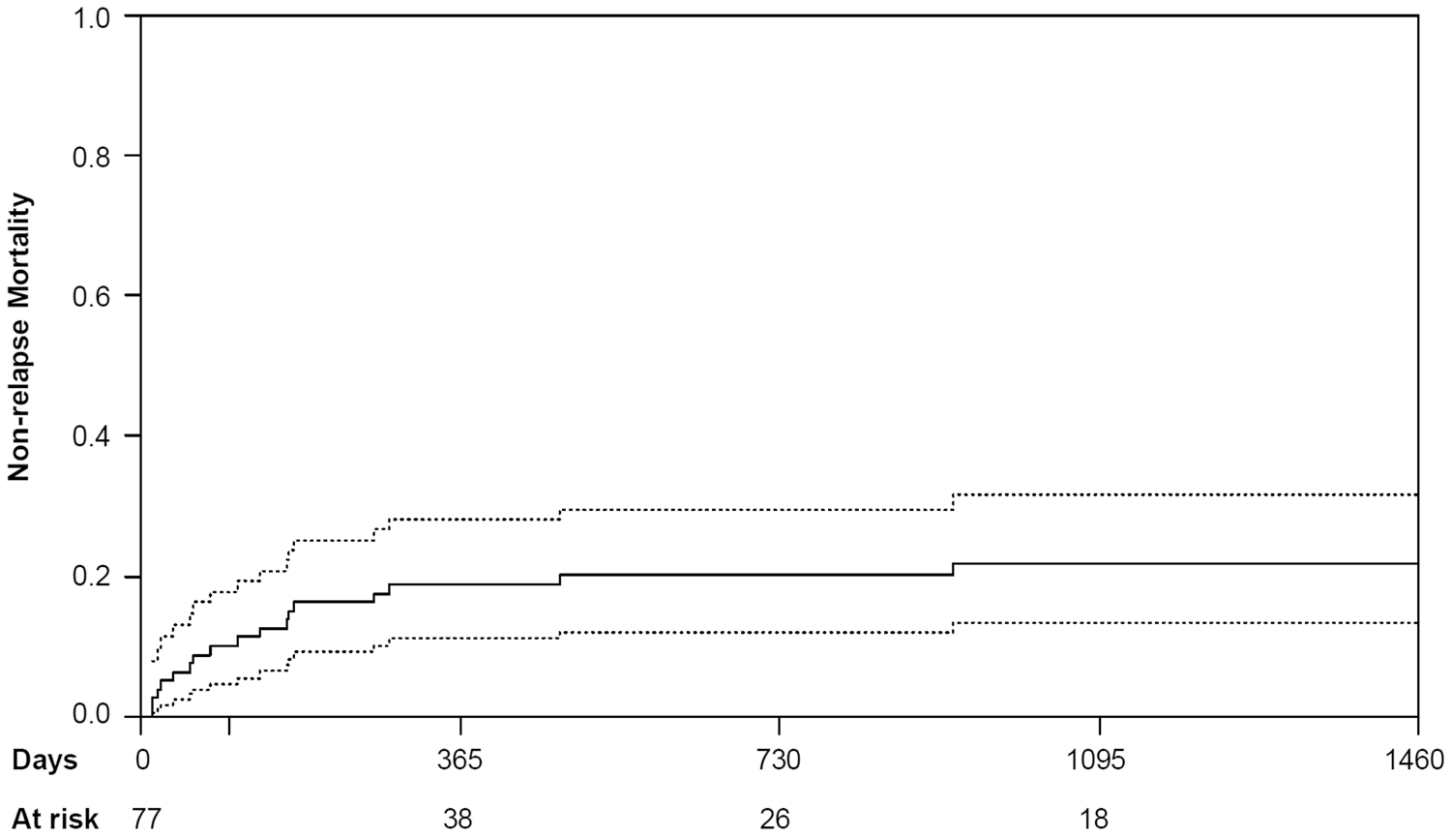
Cumulative incidence of non-relapse mortality for all patients (full line) with 95% confidence intervals (broken line)

**Fig. 3:**
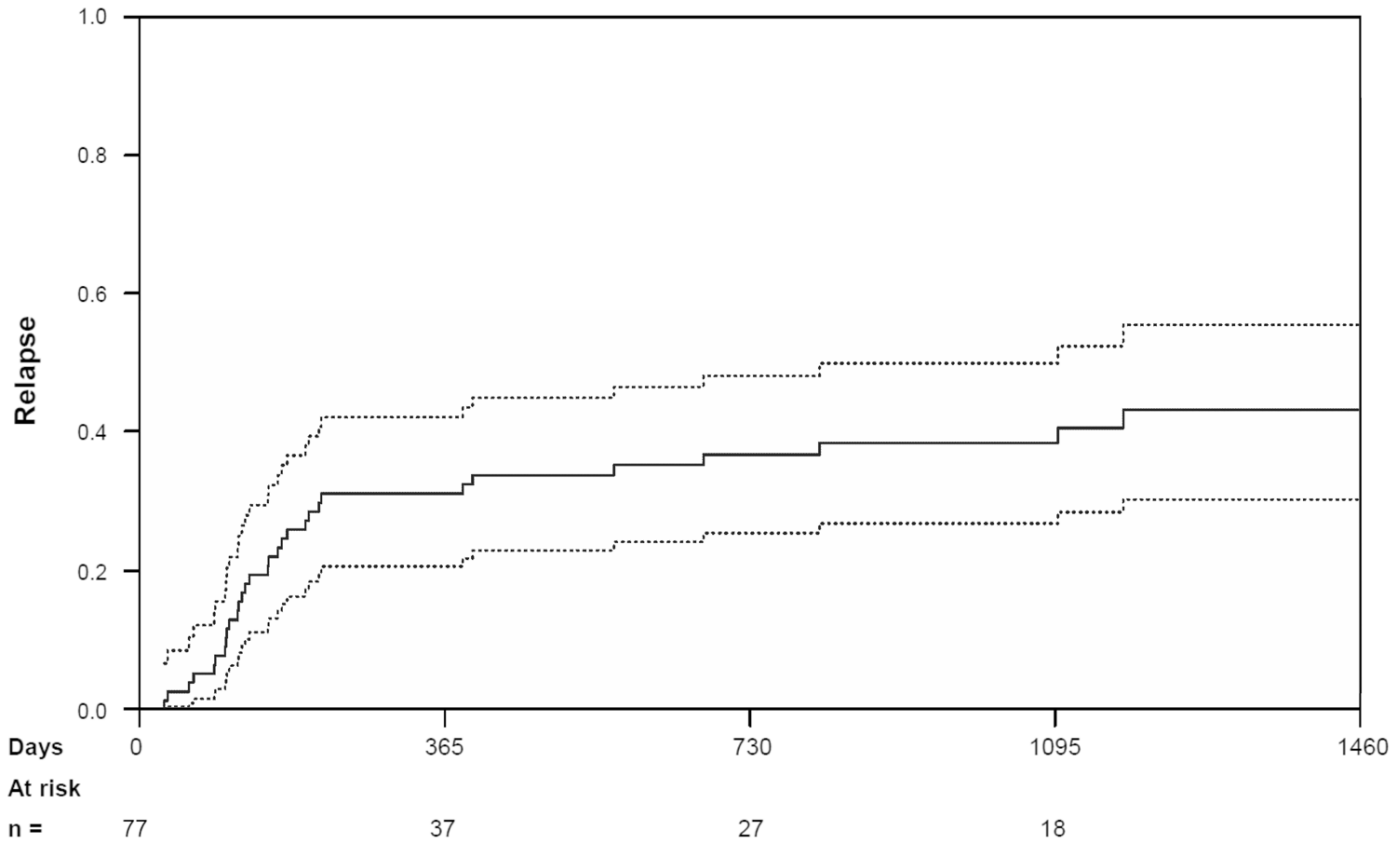
Cumulative incidence of relapse for all patients (full line) with 95% confidence intervals (broken line)

Cumulative incidences of NRM were 10.0% [95% CI (5%, 18%)) at 100 days, 18.8% (95% CI (11%, 28%)] at 1 year and 20.1% [95% CI (12%, 30%)] at 2 years (see Fig. 2). The 1-, 2- and 3-year cumulative incidences of relapse were 31.0% [95% CI (21%, 42%)], 36.8% [95% CI (26%, 48%)], and 38.5% [95% CI (27%, 50%)], respectively (see Fig. 3). The subgroup analyses for NRM and the cumulative incidence of relapse are depicted in Tables 2 and 3.

**Table 2:** Overview of the NRM rates for all patients and group analyses

Patient groups (number of transplanted patients)	Non-relapse mortality (%)			*p* value (Gray’s test)
	100 days	One-year	Two-year	
All patients (77)	10.0	18.8	20.1	
MUD (47)	12.0	16.0	16.0	0.31
MRD (20)	0.0	19.0	24.0	
MMUD (10)	20.0	20.0	30.0	
CR (35)	5.4	16.2	18.9	0.91
Non-CR (42)	14.0	20.9	20.9	
< 50 years (25)	0.0	11.0	11.0	0.077
≥ 50 years (52)	15.0	23.0	25.0	
AML patients only (58)	11.7	21.7	21.7	

*AML* acute myeloid leukemia, *CR* complete remission, *MMUD* mismatched-unrelated donor, *MRD* matched-related donor, *MUD* matched-unrelated donor

**Table 3:** Overview of the relapse rates for all patients and group analyses

Patient groups (number of transplanted patients)	Cumulative incidence of relapse (%)		*p* value (Gray’s test)
	One-year	Three-year	
All patients (77)	31.0	38.5	
MUD (47)	36.4	45.3	0.24
MRD (20)	27.0	27.0	
MMUD (10)	30.0	30.0	
CR (35)	33.0	42.5	0.96
Non-CR (42)	31.5	37.6	
< 50 years (25)	42.0	53.0	0.08
≥ 50 years (52)	27.5	31.8	
AML patients only (58)	35.0	42.5	

*AML* acute myeloid leukemia, *CR* complete remission, *MMUD* mismatched-unrelated donor, *MRD* matched-related donor, *MUD* matched-unrelated donor

### Engraftment, graft failure and chimerism

CTCAE grade IV neutropenia, leukocytopenia, and thrombocytopenia occurred in all patients. Three patients died in the pre-engraftment phase. One patient achieved neutrophil engraftment, but died before platelet engraftment was achieved. The rest achieved engraftment of neutrophils and platelets.

The day 28 cumulative incidence of engraftment for neutrophils was 85.0% [95% CI (75%, 91%)]. By day + 37, all patients had achieved successful neutrophil engraftment. The day 28 cumulative incidence of platelet engraftment was 82.5% [95% CI (72%, 89%)]. By day + 100, this had increased to 85.0% [95% CI (75%, 91%)].

The median time to neutrophil engraftment was 20 days (range 9–37 days) and to platelet engraftment 20 days (range 11–174 days). No primary or secondary failure of engraftment was documented. There were 9 cases of poor graft function with regard to neutrophil engraftment and 12 cases with regard to platelet engraftment.

The cumulative incidence of complete donor-type chimerism was 84.0% [95% CI (74%, 90%), 66 subjects] on day + 28. By day + 100, 80.0% of patients were found to have complete donor-type chimerism (55 subjects). By day + 180 this figure fell to 68.0% (41 subjects). The course of chimerism mirrored the incidence of relapse.

### Acute and chronic graft-versus-host disease

Day 100 cumulative incidences of grade I–IV, II–IV, and III–IV acute GVHD were 38.0% [95% CI (27%, 48%)], 22.0% [95% CI (13%, 33%)], and 6.0% [95% CI (2%, 14%)]. Two patients developed grade IV aGvHD of the liver. The cumulative incidence of mild to severe cGVHD at 2 years was 15.0% [95% CI (8%, 24%)]. There were three cases of severe cGvHD.

### Toxicities and adverse events

Every patient experienced the expected chemotherapy-related myelosuppression and required the transfusion of blood products following or prior to transplantation as a direct consequence of the conditioning chemotherapy. In some cases, myelosuppression was also caused by the underlying malignant condition or previous bridging or salvage therapy given prior to the start of conditioning. A detailed breakdown of recorded toxicities is depicted in Table 4.

**Table 4:** Toxicities and adverse events that occurred during conditioning up until day + 28 following transplantation, graded according to the NCI Common Terminology Criteria for Adverse Events (CTCAE) version 5.0

Adverse Event	Number of patients, *n* (%)
Oral mucositis	
Grade 1–2	14 (18)
Grade 3–4	18 (23)
Creatinine increase	
Grade 1–2	34 (44)
Grade 3–4	1 (1.3)
Sinusoidal obstruction syndrome	
Grade 3–4	2 (2.6)
Grade 5	1 (1.3)
AST/ALT increase	
Grade 1	33 (43)
Grade 2	22 (29)
Grade 3–4	17 (22)
AP increase	
Grade 1–2	45 (58)
Grade 3–4	3 (3.9)
Bilirubin increase	
Grade 1–2	52 (68)
Grade 3–4	12 (16)
Febrile neutropenia	
Grade 3–4	48 (62)
Sepsis	
Grade 3–4	32 (42)
Grade 5	2 (2.6)
Lung infection	
Grade 3–4	27 (34)
Grade 5	1 (1.3)

The diagnosis of sinusoidal obstruction syndrome (SOS) (grade 3) was suspected in two patients who suffered from progressive AML or from CML in transition to accelerated phase. A third patient suffering from progressive AML and renal insufficiency, who had received stem cells from an MMUD, likely died due to SOS (grade 5). The patients received SCT at the age of 44, 55, and 52 years from a matched related donor, except for the younger AML patient, who had an MMUD. The HCT-CI was 3, 0 and, 0, respectively (Sorror et al. 2005). All three patients received the same fludarabine containing conditioning therapy as well as the same GvHD prophylaxis and none of them had received gemtuzumab ozogamicin, which is known to increase the risk of SOS (Corbacioglu et al. 2019).

Further adverse events included hemorrhagic colitis (1), neutropenic colitis (1), post-transplant lymphoproliferative disease (1), pericardial effusion with hemodynamic relevance (2), non-ST-segment elevation myocardial infarction (NSTEMI) (1), pulmonary embolism (1), and cardiac decompensation (2). Two patients developed a secondary malignancy. The first developed gallbladder cancer 3 years following an MMUD transplantation for AML. The second developed a ductal carcinoma in situ just over two-and-a-half years following an MUD transplantation for CML.

### Discussion

This retrospective dual center study evaluated the outcomes of patients with AML, MDS or MPN and high risk of relapse following conditioning with Treo/Flu/AraC prior to allogeneic SCT. The addition of cytarabine combined with the higher dose of treosulfan (14 g/m^2^ vs. 10 g/m^2^ as used in Beelen et al. 2020) aimed to enhance and optimize the antileukemic activity of this regimen.

To date, the majority of prospective Treo/Flu studies have selected AML patients in CR or MDS/CML patients with a low relapse risk (Beelen et al. 2020; Casper et al. 2012; Ruutu et al. 2011; Michallet et al. 2012). This complicates cross-trial comparisons with the patients reported here. The patients in the current study either suffered from active disease or were in CR with high risk of relapse due to: (1) cytogenetic factors, (2) sAML, (3) post-salvage chemotherapy or (4) high-risk MPN (see Table 1). Many existing studies do not specify if salvage chemotherapy was required to achieve CR, instead relying on the statement, ‘indication for allogeneic SCT according to institutional policy’. This description potentially hides a wide spectrum of differently responsive leukemic blasts and further hinders cross-trial comparisons.

In the current study, 54% of patients were not in CR at the time of transplantation, with a median blast count of 22.6%. Secondary AML was diagnosed in 32% of patients. In the univariate analysis performed here, there was no difference in the incidence of relapse between patients transplanted in CR and non-CR. This suggests that the patients transplanted in CR were, despite their status, still at a very high relapse risk. Taking these factors into consideration, RFS and OS in the current study were acceptable and comparable to the observed outcomes in the Treo/Flu studies examining higher-risk patient populations (sAML or active disease at SCT) (Kroger et al. 2006; Nagler et al. 2017).

The FLAMSA-RIC protocol was designed to reduce AML disease burden prior to transplantation and is often used in patients with active disease. In a retrospective analysis of 60 AML patients (CR 57%, active disease 43%) conditioned with FLAMSA-RIC, Krejci et al*.* (2013) also observed RFS and OS outcomes comparable to the ones presented here.

The critical period for NRM are the first 2 years after SCT, in line with the findings of long-term follow-up studies of post-transplant AML patients (Shimoni et al. 2016; Socie et al. 1993). The NRM after conditioning with Treo/Flu/AraC was 20.1% at 2 years. Of note, the addition of cytarabine did not worsen NRM and compared well with previous studies (Casper et al. 2010; Ruutu et al. 2011) in our heavily pre-treated patient population. Besides cytogenetic risk stratification, response to prior treatment reflects the biological behavior of the patient’s disease. As decidedly more patients in this study were not in CR at SCT, NRM rates are higher compared to the studies conducted by Gyurkocza et al. (2014) and Deeg et al. (2018).

However, our results are comparable to a recent study from the EBMT demonstrating lower 2-year NRM rates for patients with active AML treated with FLAMSA-RIC (7% vs. 16% in Bu/Cy, 19% in Cy/TBI and 18% in FLAMSA-TBI) and higher 2-year OS (50% vs. 33% in Bu/Cy, 34% in Cy/TBI and 36% FLMASA-TBI). The 2-year cumulative incidence of relapse was 51% for Cy/TBI, 56% for Bu/Cy, 55% for FLAMSA-TBI and 53% for FLAMSA-RIC (Rodriguez-Arboli et al. 2020). The 2-year cumulative incidence of relapse (36.8%) reported here was acceptable for the high-risk patient population. Two-year relapse rates in the Treo/Flu studies ranged from 16%, in an analysis of 45 patients with primary MDS (Ruutu et al. 2011), to 34% in a study of 75 patients with AML transplanted in CR (Casper et al. 2012). Two-year cumulative incidence of relapse in patients transplanted with FLAMSA-RIC ranged from 22.8% for high-risk AML patients transplanted in CR (Malard et al. 2017) to 52% for patients with exclusively primary refractory or relapsed AML (Schneidawind et al. 2013).

The presence and severity of aGvHD and/or cGvHD is also associated with a reduced risk of AML, CML and MDS relapse (Patel et al. 2016; Stern et al. 2012). The incidence of II–IV aGvHD is roughly around 35–50% in all recipients of allogeneic HSCT (Jacobsohn and Vogelsang 2007). The cumulative incidence of grade II–IV aGvHD in the present study (22%) is low relative to this figure, but was comparable to that seen in Treo/Flu and FLAMSA-RIC studies (Remberger et al. 2017; Pfrepper et al. 2016). Severe aGvHD was only recorded in 6% of patients. In addition to the low rate of aGvHD, the cumulative incidence of cGvHD after Treo/Flu/AraC-based conditioning was low and potentially contributed to the observed relapse rate.

The cumulative incidence of mild to severe cGvHD at 2 years in the current study was 15%. This figure is low compared to the results of the numerous Treo/Flu and FLAMSA-RIC studies, where the incidence ranged from 24% (Baronciani et al. 2008) to 72% (Hilgendorf et al. 2011). The finding might be explained by the immunosuppressive characteristics of treosulfan, which has been shown to result in less proinflammatory cytokine release than busulfan or cyclophosphamide in a mouse model (Sjoo et al. 2006). However, evidence against this theory has been published by Beelen et al. who revealed similar rates for aGvHD and cGvHD, when comparing allogeneic SCT with either Treo/Flu or Bu/Flu conditioning (Beelen et al. 2020). Attributing the low rates of GvHD found in our study solely to the immunosuppressive effects of treosulfan seems unlikely. Comparing conditioning with cyclophosphamide and TBI with or without cytarabine also found no differences in the incidence of GvHD, effectively excluding the influence of cytarabine to explain the discrepancy (Arai et al. 2015).

The engraftment and chimerism results show that the Treo/Flu/AraC regimen performs well against other regimens in preparing the host BM for the engraftment and proliferation of donor stem cells. There was no case of primary or secondary graft failure.

Three cases of SOS (4%) were reported here. Busulfan-based conditioning regimens are associated with an increased risk of developing SOS (Dix et al. 1996). Early phase II Treo/Flu studies did not report any cases of SOS (Casper et al. 2012, 2004b), suggesting that the use of treosulfan confers a lower risk of this complication. A later retrospective study found an incidence of 2.2% and two deaths caused by SOS under Treo/Flu conditioning (Nagler et al. 2017). Having identified patient- and transplant-related risk factors for patients with SOS, the results of this study suggest that the addition of hepatically metabolized cytarabine to the regimen does not dramatically increase the risk of developing SOS.

A grade 3/4 rise in bilirubin and AST/ALT levels exceeded 10%, but on the whole organ toxicities were mild and reversible. Grade 3/4 infections, both sepsis and lung infections exceeded an incidence of 30%. This was as a consequence of the duration and severity of marrow suppression; however, the incidences did not exceed those previously observed in other Treo/Flu or FLAMSA-RIC studies (Ruutu et al. 2011; Krejci et al. 2013; Casper and Freund 2004). Nevertheless, the findings of this study have to be interpreted with caution and the retrospective character and the highly heterogeneous patient population should be kept in mind.

In summary, Treo/Flu/AraC is a feasible, effective, and safe regimen, which can be used to condition patients unsuitable for myeloablative protocols at high risk of disease relapse prior to allogeneic SCT. The incidence of acute and chronic GvHD is low compared to that seen with the FLAMSA-RIC protocol. Non-hematological toxicities were mild and reversible and RFS and OS were comparable to similar patient groups conditioned with Treo/Flu or FLAMSA-RIC. Compared to the FLAMSA-RIC protocol, Treo/Flu/AraC has a shorter conditioning period with the potential for a reduced duration of hospital stay and shorter neutropenic phase. Large, prospective, and randomized controlled trials are required to verify these findings and identify patients who would benefit most from this regimen. The idea of disease-specific conditioning protocols, such as cytarabine for AML and melphalan for myeloma, using treosulfan as a backbone should be investigated in the era of personalized medicine.

## Supplementary Information

Below is the link to the electronic supplementary material.

Supplementary file1 (DOCX 18 KB)

## Data Availability

The datasets generated during and/or analyzed during the current study are available from the corresponding author on reasonable request.
