# Supplementary material for: Combination of treosulfan, fludarabine and cytarabine as conditioning in patients with acute myeloid leukemia, myelodysplastic syndrome and myeloproliferative neoplasms
Source: J Cancer Res Clin Oncol. 2021 Oct 21;148(10):2599–609. doi: 10.1007/s00432-021-03836-8 (PMC9470667; doi:10.1007/s00432-021-03836-8)
Supplement: Supplementary file 1 — Supplementary file1 (DOCX 18 KB) [file 432_2021_3836_MOESM1_ESM.docx]

**Combination of treosulfan, fludarabine and cytarabine as conditioning in patients with acute myeloid leukemia, myelodysplastic syndrome and myeloproliferative neoplasms**

Running title: Treosulfan/Fludarabine/Cytarabine conditioning

Samantha O‘Hagan Henderson^1*^, Jochen J. Frietsch^2*^, Inken Hilgendorf^2^, Andreas Hochaus^2^, Claus-Henning Köhne^1^, Jochen Casper^1^

* the both authors contributed equally

^1^Universitätsklinikum Oldenburg, Klinik für Innere Medizin II, Onkologie und Hämatologie, Oldenburg, Germany

^2^Universitätsklinikum Jena, Klinik für Innere Medizin II, Abteilung für Hämatologie und Internistische Onkologie, Jena, Germany

**Table S1:** Overview of the RFS rates for all patients and group analyses

| **Patient Groups (number of transplants/patients)** | **Relapse-Free Survival (%)** | | | ***P*-value**  **(log-rank test)** |
| --- | --- | --- | --- | --- |
|  | One-year | Two-year | Three-year |  |
| All transplanted patients (77) | 49.4 | 41.7 | 37.6 |  |
| MUD (47)  MRD (20)  MMUD (10) | 48.9  55.0  40.0 | 39.7  49.5  40.0 | 37.0  41.3  40.0 | 0.74 |
| CR (35)  Non-CR (42) | 55.1  50.2 | 48.2  45.5 | 43.8  43.6 | 0.992 |
| <50 years (25)  ≥50 years (52) | 48.0  50.0 | 40.0  43.9 | 36.0  41.0 | 0.966 |
| AML patients (58) | 43.3 | 38.1 | 33.5 |  |

AML, acute myeloid leukemia; CR, complete remission; MMUD, mismatched unrelated donor; MRD, matched-related donor; MUD, matched-unrelated donor.

**Table S2:** Overview of the OS rates for all patients and group analyses

| **Patient Groups (number of transplanted patients)** | **Overall Survival (%)** | | | ***P*-value**  **(log-rank test)** |
| --- | --- | --- | --- | --- |
|  | One-year | Two-year | Three-year |  |
| All patients (77) | 59.3 | 49.3 | 45.4 |  |
| MUD (47)  MRD (20)  MMUD (10) | 56.8  70.0  48.0 | 49.9  48.1  48.0 | 46.7  40.1  48.0 | 0.95 |
| CR (35)  Non-CR (42) | 65.6  54.3 | 53.3  46.2 | 45.1  46.2 | 0.996 |
| <50 years (25)  ≥50 years (52) | 58.1  60.3 | 54.2  47.4 | 44.4  44.5 | 0.785 |
| AML patients only (58) | 56.5 | 48.9 | 43.5 |  |

AML, acute myeloid leukemia; CR, complete remission; MMUD, mismatched unrelated donor; MRD, matched-related donor; MUD, matched-unrelated donor.
